# Three new species of *Grouvellinus* Champion, 1923 from Maliau Basin, Sabah, Borneo, discovered by citizen scientists during the first Taxon Expedition (Insecta, Coleoptera, Elmidae)

**DOI:** 10.3897/zookeys.754.24276

**Published:** 2018-04-30

**Authors:** Hendrik Freitag, Clister V. Pangantihon, Iva Njunjić

**Affiliations:** 1 Ateneo de Manila University, Department of Biology, School of Science & Engineering, Loyola Heights, Quezon City 1101, Philippines; 2 Taxon Expeditions, Rembrandtstraat 20, 2311 VW, Leiden, Netherlands; 3 Naturalis Biodiversity Center, Vondellaan 55, 2332 AA, Leiden, Netherlands

**Keywords:** André Kuipers, Leonardo DiCaprio, new species, riffle beetle, taxon expeditions, taxonomy, Quest magazine

## Abstract

Further results are presented of the first field course at Maliau Basin, Malaysian Borneo organized by Taxon Expeditions, an organization which enables citizen scientists to be directly involved in taxonomic discoveries. Three new species of the aquatic beetle genus *Grouvellinus* Champion, 1923, namely *G. leonardodicaprioi* **sp. n.**, G. *andrekuipersi* **sp. n.**, and *G. quest* **sp. n.** were collected jointly by the citizen scientists and taxonomists during the fieldwork in Maliau Basin. Material was mainly sampled from sandstone bottom rocks of blackwater streams at altitudes between 900 m and 1,000 m using fine-meshed hand-nets. The genus is widely distributed in the Oriental and Palearctic regions, but these are the first records from the island of Borneo.

## Introduction

During the first biodiversity discovery field course for citizen scientists, termed “taxon expedition” (Schilthuizen et al. 2017), participants collected six new species for science from Maliau Basin, Malaysian Borneo. Three of them, litter-dwelling Coleoptera, were recently described jointly by taxonomists and citizen scientists (Schilthuizen et al. 2017). The taxonomic treatments for the other three species, all belonging to the genus *Grouvellinus* (Elmidae), are provided here. Names of the new species were selected in two ways: during a naming ceremony in Maliau Basin by the course participants and staff of the Maliau Basin Studies Centre; and in an online public contest organized by the Dutch science program De Kennis van Nu (2017).

Citizen Science initiatives in biodiversity research have become increasingly popular with an estimated annual growth rate of 10% in number of projects launched (Theobald et al. 2015). They generate important scientific information, especially in terms of biodiversity monitoring (Chandler et al. 2017), planning and management of ecosystems and protected areas (e.g., Pollock and Whitelaw 2005), species distribution (e.g., Suprayitno et al. 2017), and conservation (McKinley et al. 2017), among other fields. Some of them run successfully already for decades and have involved thousands of citizens (e.g., Florida Lakewatch 2017, EarthWatch Institute 2018). However, it is rather rare that the results are published in peer-reviewed journals, which is considered, last but not least, an important quality control for scientific data. Theobald et al. (2015) concluded that only 12% of several hundred citizen science projects screened refer to peer-reviewed published results, although it is possible that more citizen scientist data were published, but not clearly indicated as such.

Conrad and Hilchey (2011) point out that “the nature of citizen science implies that in many cases, the work being undertaken is not documented in traditional journal articles, although there certainly are exceptions”. Our taxon expeditions are in fact such an exception, as the ultimate goal is to publish the descriptions of the new species that have been discovered and tentatively named by our participating citizen scientists. We also encourage their direct involvement as co-authors in the process of the verbal species’ descriptions and scientific illustration of diagnostic characters (although not embraced in this paper, but see Schilthuizen et al. 2017). The focus on publishing species discoveries by citizen scientists makes our initiative unique, since such approaches are only seen in student volunteer funded programs before (e.g., Opwall 2018).

Since basic research on invertebrates (including taxonomy and faunistics) is relatively infrequent and underfunded (Cardoso et al. 2011), the valued financial contribution of the participating citizen scientists enables taxonomic explorations in key biodiversity areas which are very unlikely being supported by any conventional science funding agency.

This paper deals with three new species of the genus *Grouvellinus* Champion, 1923. The genus, named in honour of the French coleopterologist Antoine Henri Grouvelle (1843–1917), comprises small to medium-sized riffle beetles (Elmidae) of dark (usually black), or rarely cupreous colour and is distributed in the Oriental and Palaearctic regions from Samos (Greece) in the west up to Japan in the East and Java (Indonesia) in the south (Jäch et al. 2016). It is also recorded from the neighbouring Philippines where several species await formal description (Freitag et al. 2016). Forty species are known to science (Jäch et al. 2016, Bian and Jäch 2018), of which seven are endemic to Indonesia and one, *Grouvellinus bishopi* Jäch, 1984, to Peninsular Malaysia. Since there are no previously published records from the island of Borneo, our data extend the known distributional range of *Grouvellinus* to the largest Asian island. *Grouvellinus* species are sometimes hardly distinguishable by external morphological characters, but the differences in their aedeagi are diagnostic.

**Figure 1.**
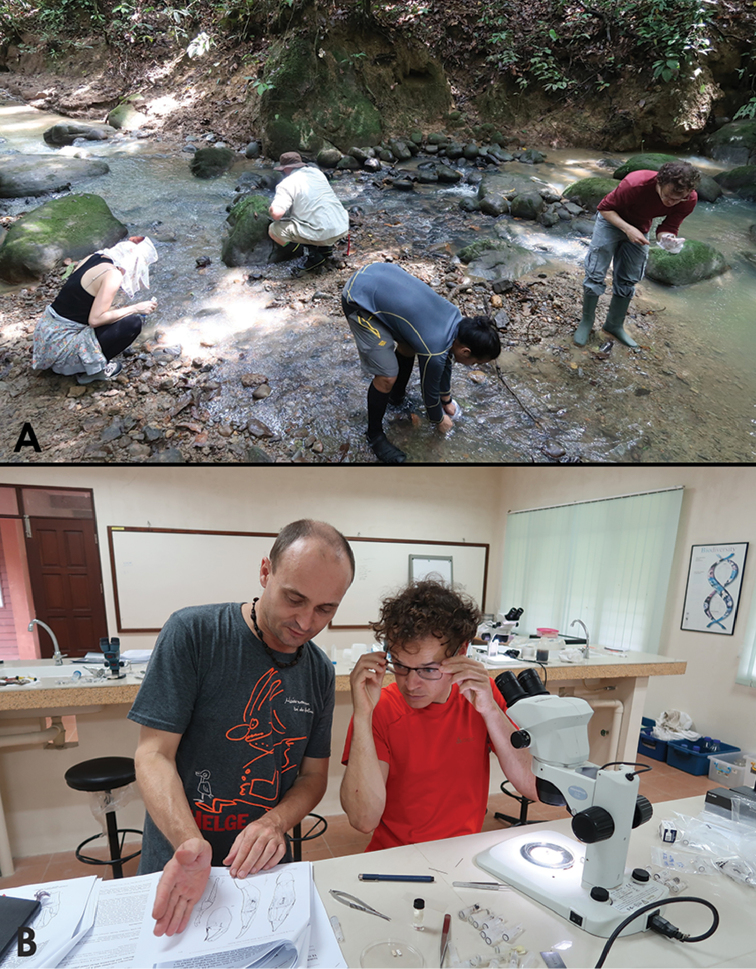
**A** Citizen scientists on the first taxon expedition to Maliau Basin, Malaysian Borneo, performing microhabitat sampling **B** The first author (on the left) instructing journalist Paul Serail of QUEST magazine (on the right) on the identification of riffle beetles.

## Materials and methods

The specimens were collected by taxon expedition participants and instructors using small-meshed hand-nets and preserved in 96% ethanol. After return to the field station, participants sorted the collected specimens to genus level under a Nikon SMZ445 stereomicroscope with 20 × oculars (allowing magnification up to 70 ×) and with the help of provided taxonomic literature. Microforceps, insect pins, and the same optical equipment were subsequently used for the dissection of specimens and the detailed material examination. This step was carried out by the participants only for morpho-species of which larger series were available, while the instructors handled single records of new species to limit damage through unexperienced handling of potential holotypes. Diagnostic characters of the external habitus and the genitalia were briefly recorded by the participants.

Photographs of dissected parts were taken from temporary slides in lactic acid under an Olympus CX compound microscope. Preliminary photographs of the dorsal habitus of entire specimens (as used in the public species-naming contest) were obtained by the participants using a Canon EOS 500D with MP-E 65 mm lens attached to a Kaiser stand with vertical micro-adjustment drive. Series of vertical photographs series were taken and layers were subsequently stacked using CombineZP software.

After the actual field course, CorelDRAW Version 10.0 software was used by the authors to compile digital line drawings. Additional high-quality photographs were taken under a Zeiss Axio zoom V 16 microscope with Canon 5D SLR camera using diffuse LED lighting. Images were captured at various focus layers and subsequently stacked using the Zerene Stacker software.

Morphological terminology used herein mainly follows the Elmidae chapter of the Handbook of Zoology/Coleoptera (Kodada et al. 2016). Elytral striae (rows of punctures) and intervals are numbered as actually visible (from suture to lateral rim) ignoring presumable fusion of seventh and eighth striae (comp. Jäch 1984).

The following abbreviations were used:

**a.s.l.** above sea level (elevation)

**CL** calculated length (PL + EL)

**EL** elytral length, measured along the elytral suture from basis to apex

**EW** maximum elytral width

**HW** head width, including eyes

**ID** interocular distance

**PW** maximum pronotal width (at posterior portion)

**PL** pronotal length

All collected material is deposited at the Borneensis Coleoptera Collection (**BOR/COL**) of the Institute for Tropical Biology and Conservation, Universiti Malaysia Sabah, Kota Kinabalu. Previously undetermined specimens of the Coleoptera collections of the BOR/COL repository and the Natural History Museum Vienna, Austria (**NMW**) were checked by the first author for conspecific material which was designated as paratype material, in order to extend the type series and distributional knowledge. We additionally refer to some material from the Sabah Parks Museum, Kinabalu Park Headquarters, Malaysia (**SP**).

## Taxonomy

### Genus *Grouvellinus* Champion, 1923

#### Grouvellinus leonardodicaprioi sp. n.

Taxon classificationAnimaliaColeopteraElmidae

http://www.zoobank.org/F5E77C1D-1938-417E-A94D-D2624ECEAB3A

Figures 2 , 5 , 6

##### Type locality.

Malaysia, Sabah (Eastern Borneo Island), Maliau Basin, upstream Giluk Falls, ca. 4°44'49"N, 116°52'38"E, ca. 950 m a.s.l. (Fig. 12A)

##### Type material.

**Holotype** ♂ (BOR/COL): “MALAYSIA: Sabah: Maliau Basin: \ upstr. Giluk Falls; bottom rock, run; \ ca. 4°44'49"N, 116°52'38"E, ca.950m a.s.l. \ 01.X.2017, leg. I. Njunjić, CV. Pangantihon, P. Serail (GilF3g)”; terminal parts of abdomen incl. aedeagus glued separately; right foreleg, left protarsus incl. parts of protibia and left antenna lacking; right elytral apex slightly damaged.

##### Etymology.

The new species is named in honour of the actor Leonardo DiCaprio to acknowledge his inspiring work in promoting environmental awareness and bringing the problems of climate change and biodiversity loss into the spotlight. The species name was selected during a naming ceremony at Maliau Basin Studies Centre on 6 October 2017, in which expedition participants as well as a large number of field centre staff and porters took part.

##### Description.

*Body* obovate, 2.97 mm long (CL), 1.60 mm wide (EW), 1.9 times as long as wide (CL/EW).

**Figures 2–4.**
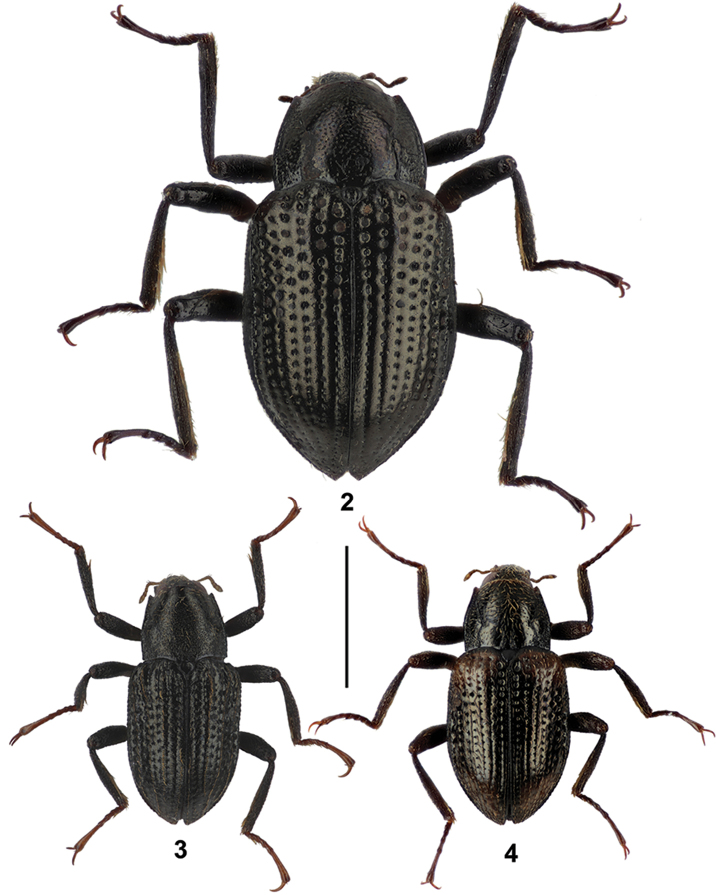
Habitus of the new *Grouvellinus* species collected from the Maliau Basin: **2** *G. leonardodicaprioi* sp. n. (image of holotype male partly complemented) **3** *G. quest* sp. n. (paratype male from ‘NepC3g’) **4** *G. andrekuipersi* sp. n. (paratype male from ‘NepC3g’). Scale bar: 1 mm.

**Figure 5.**
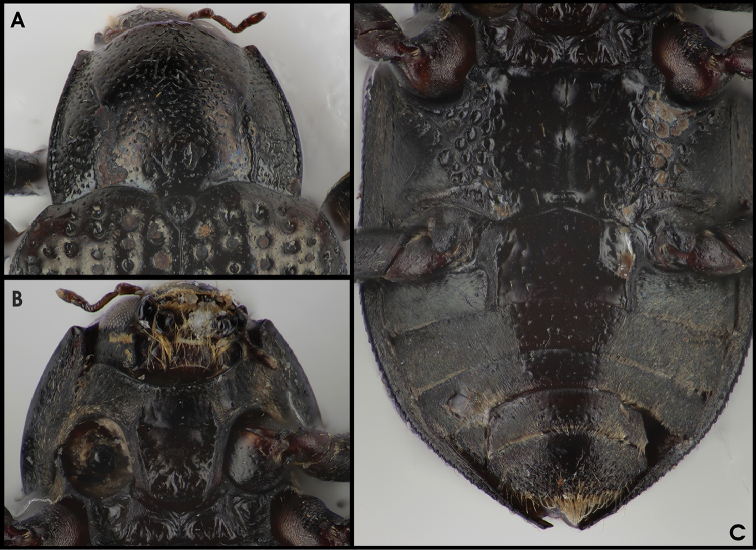
*Grouvellinus leonardodicaprioi* sp. n. (holotype male): **A** anterio-dorsal aspect with pronotum **B** anterio-ventral aspect with prosternum **C** posterio-ventral aspect with meso-, metaventrite, and ventrites 1–5.

*Dorsal colouration* (Figs 2, 5A) black with slight metallic lustre; claws and antennae dark brown; pubescence yellow. Ventral side (Figs 5B, C) very dark brown. Plastron pubescence shiny golden.

*Head* 0.65 mm wide (HW); ID 0.27 mm; partly retractable; labrum with dense fringes of moderately long setae, punctures very small and dense; frons and clypeus moderately pubescent, setae moderately long, punctures small and moderately dense; intervals almost flat and glabrous. Frontoclypeal suture distinct, slightly concave. Eyes moderately protruding. Antennae genus-typical, slightly shorter than HW.

*Pronotum* (Fig. 5A) 0.85 mm long (PL), 1.12 mm wide (PW), distinctly wider than long (PL/MW), widest posterior 0.25, distinctly narrower than elytra, anteriorly distinctly attenuate; anterior margin convex; median carina absent, distinct sublateral carinae present posterior 0–0.3; oblique impressions very shallow; laterobasal impression very shallow, indistinct; pronotal disc moderately vaulted, densely punctate; punctures moderately big and shallowly impressed; interstices glabrous and flat; setae comparably short; pronotal impressions appearing rugose by irregularly enlarged punctures. Hypomeron rugose, increasingly pubescent (plastron) ventrad.

*Prosternum* (Fig. 5B) very short, lateral portions with very dense, fine pubescence (plastron); median portion and process glabrous; prosternal process distinctly partitioned and elevated from remaining prosternum, short, distinctly wider than long, posteriorly broadly rounded; margins conspicuously fringed; sub-posteriolateral portion slightly impressed, rugose.

*Scutellum* (Fig. 5A) elongate sub-cordiform, posteriorly impressed, glabrous.

*Elytra* (Figs 2, 5A) roundly elongate, strongly convex dorsally, 2.31 mm long (EL), ca. 1.4 times as long as wide (EL/EW); apices separately conically pointed in posterior 0.05, with eight to nine (most lateral row partly divided anterior 0.3–0.7) longitudinal rows of punctures (striae); striae moderately (anterior portion) to inconspicuously (posterior portion) impressed; punctures of rows 2 and 3 dissolved beyond anterior 0.8; remaining punctures somewhat regularly arranged (except for most lateral row), but distinctly varying in size and degree of impression: much larger (2–6 times of intervals) and deeply impressed medio-basally, small (0.1–0.3 times of intervals) and shallowly impressed sub-apically; interstices glabrous; intervals 7 and 8 with finely crenulate carinae approx. from basal 0.1 to 0.9; carinate interval 8 extending as “shoulder crest” basad up to pronotal angle; intervals 1–3 slightly broadly elevated basal 0.1–0.2; at least intervals 1, 3, 5, 7, 8 with rows of scattered setae (most of them presumably broken off in the specimen examined); lateral elytral margin serrate and with row of setae.

*Mesoventrite* (Fig. 5C) with two pairs of moderately deeply impressed grooves, one sub-rectangular pair behind procoxae and another sub-trapezoidal pair medially behind prosternal process.

*Metaventrite* (Fig. 5C) disc glabrous, with longitudinal impression along median suture, medioanteriorly impressed; anterior angles (bordering mesocoxae) process-like elevated; lateral metaventrite portions with large irregular punctures or impressions; their interstices rugulose; most lateral portion (approx. between hind coxae and shoulder) with dense plastron pubescence.

*Abdominal ventrites* (Fig. 5C). Ventrite 1 with pair of longitudinal carinae between glabrous disc and densely pubescent lateral portions (plastron); broad lateral portions of ventrites 1–4 and almost entire ventrite 5 densely covered with plastron (Fig. 5C); median glabrous portions with sparse punctures and small setae.

*Legs* (Fig. 2) slightly shorter than body, hind leg longest; hind tibiae slightly broader than those of the other legs; tibia longer than tarsus and femur in all legs; parts of coxae, proximal femora and entire inner (ventral) face of tibiae and femora moderately densely covered with short adpressed setae; outer (dorsal) edge of femora and tibiae and inner (ventral) edge of tarsomeres 1–4 with fringes of long trichoid setae; remaining portions with scattered short setae; distal portion of tibiae and inner edge with longitudinal rows of robust setae; apex of tibiae additionally with pair of spines.

**Figure 6.**
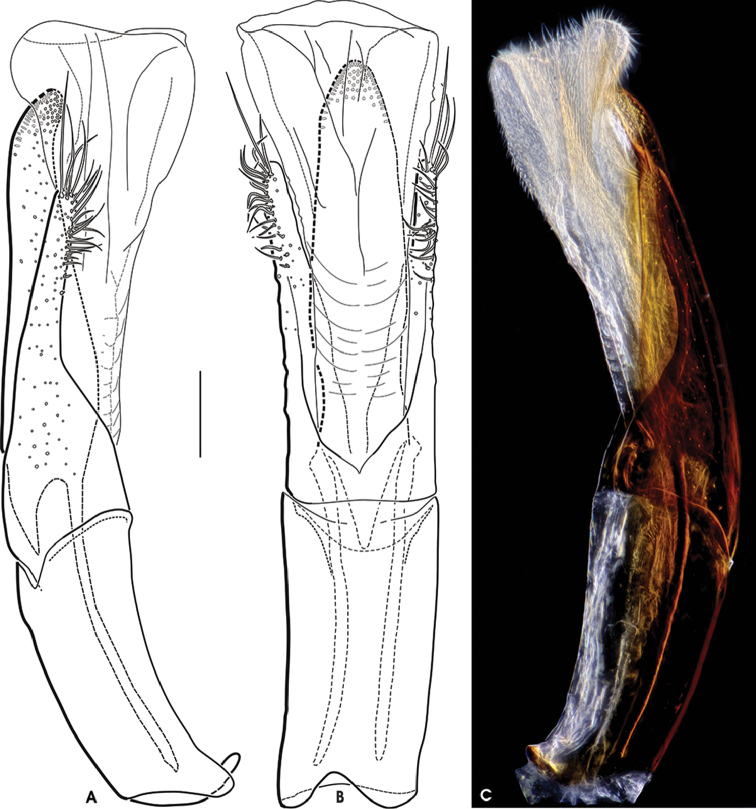
*Grouvellinus leonardodicaprioi* sp. n. (holotype male): **A** aedeagus in lateral view (spines of ventral sac omitted) **B** aedeagus in ventral view (spines of ventral sac omitted) **C** microscopic photograph of aedeagus in lateral view optically emphasizing the dense distribution of internal and external spines of the ventral sac. Scale bar: 0.1 mm.

*Aedeagus* (Fig. 6A–C) ca. 840 μm long, ca. 180 μm wide. Phallobase slightly asymmetrical basally, reaching basal 0.41 of total aedeagus length. Median lobe almost four times as long as wide, distinctly overreaching parameres, evenly slightly conical towards round apex. Ventral sac large, apically inflated beyond apex (presumably more inflated than in regular position), internal surface entirely densely covered with moderately short and thin spines, outer surface also covered with such spines up to the level of the parameres (presumably because more inflated than in regular position). Parameres distinctly shorter than median lobe, apices evenly rounded (lateral view), conical from insertion to half-length and very slender in apical half in lateral view, with approx. 20 trichoid setae in apical fourth of ventrolateral margin; most apical setae distinctly longer than all others.

*Male sternite IX* (‘spiculum gastrale’) with posterior margin rounded and entirely fringed with a broad, distinctly sclerotized margin; paraprocts closely attached to posterior portion, sub-equally long, not reaching apical margin; median strut broken and not examined.

##### Female and larva.

Unknown.

##### Differential diagnosis.

By its unusually large size, *Grouvellinus leonardodicaprioi* sp. n. resembles *G. hercules* Jäch, 1984 from Nepal, which also shares some other characters with the new species (only 7^th^ and 8^th^ elytra interval crested, margins of prosternal process fringed, elytral apices pointed), but *G. leonardodicaprioi*, sp. n. can be distinguished by the slenderer elytra, the fully glabrous (in between punctures) and not elevated median pronotum, the shallower elytral striae, as well as by its conspicuously varying aedeagus with broad main piece which distinctly overreaches the evenly rounded paramere tips (vs. very slender main piece only slightly overreaching the conically tapered paramere tips in *G. hercules*). The large size and other characters mentioned above also allow clear distinction from the species described below and any known congeners from Malaysia and Indonesia.

##### Distribution.

This species is only known from the type locality, the Giluk Falls of the upper Maliau Basin, Sabah (Figs 11, 12A).

#### Grouvellinus quest sp. n.

Taxon classificationAnimaliaColeopteraElmidae

http://www.zoobank.org/8998CB72-A2AD-4D43-AEA0-5382BD450D93

Figures 3 , 7 , 8

##### Type locality.

Malaysia, Sabah (Eastern Borneo Island), Maliau Basin, Creek east of ‘Nepenthes Camp’, ca. 4°44'57"N, 116°52'45"E, 1000 m a.s.l. (Fig. 12B).

##### Type material.

**Holotype** ♂ (BOR/COL): “MALAYSIA: Sabah: Maliau Basin: \ Creek E Nepenthes Camp; bottom rock, \ run; ca. 4°43'57"N, 116°52'45"E, ca. 1000m a.s.l. \ 01.X.2017, leg. I. Njunjić, P. Serail, C. de Groot (NepC3g)”; terminal parts of abdomen incl. aedeagus glued separately on entomological cards. **Paratypes**: 3 ♂, 4 ♀ (BOR/COL): same data as holotype; 3 ♂, 3 ♀ (BOR/COL) “MALAYSIA: Sabah: Maliau Basin: \ Giluk River; Cryptochorinae water plants, \ run; ca. 4°44'36"N, 116°52'21"E, ca. 980m a.s. \ l.01.X.2017, leg. I. Njunjić, H. Freitag, L. Seip, P. Piccoli (GilR2r)”; 1 ♂, 5 ♀, (BOR/COL) “MALAYSIA: Sabah: Maliau Basin: \ upstr. Giluk Falls; bottom rock, run; \ ca. 4°44'49"N, 116°52'38"E, ca. 950m a.s.l. \ 01.X.2017, leg. I. Njunjić, CV. Pangantihon, P. Serail (GilF3g)”; 1 ♂, 1 ♀ (NMW) “BRUNEI: Mt. Pagon \ 61 ARIF3 \ 4°20'35.8"N, 115°15'40.6"E \ 5.VI.2012 \ leg. K. Baker”; 5 ♂, 4 ♀ (NMW) “BRUNEI: Muara, Mukin Kilanas, \ Wasai Kendal Fall; sandy lowland creek; \ sec. forest; c.10m asl, c. 4°52'N, 114°53'E \ 15.6.1997 leg. Mendoza (3)”.

##### Etymology.

The species epithet refers to the English noun ‘quest’ (search, aspiration) in reference to the intense search for riffle beetles at Maliau Basin which was a big quest for the citizen scientists involved in the project. Additionally, the new species is named for the Dutch popular science magazine QUEST of which journalist Paul Serail joined the first taxon expedition. The word is used as a noun in apposition.

##### Description.

*Body* elongate obovate, 1.5–1.8 mm long (CL), 0.73–0.85 mm wide (EW), 2.1 times as long as wide (CL/EW).

**Figure 7.**
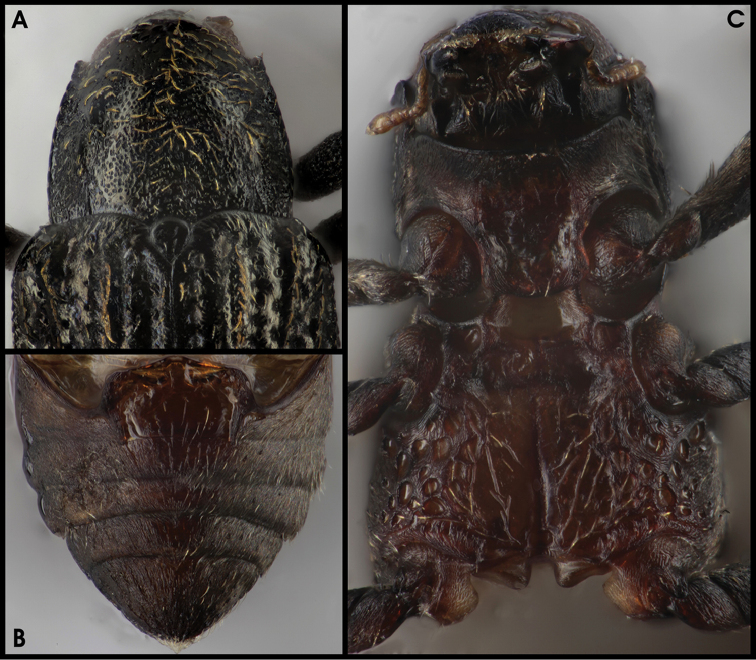
*Grouvellinus quest* sp. n. (paratype males from NepC3g): **A** anterio-dorsal aspect with pronotum **B** posterio-ventral aspect with ventrites 1–5 **C** anterio-ventral aspect with prosternum, meso- and metaventrite.

*Dorsal colouration* (Figs 3, 7A) predominantly black; tarsi, antennae, and maxillary palps reddish dark brown; pubescence yellow. Ventral side (Fig. 7B, C) reddish dark brown.

*Head* 0.35–0.37 mm wide (HW); ID 0.14–0.21 mm; partly retractable; frons, clypeus, and labrum sparsely pubescent, slightly denser laterally; punctures small and scattered; intervals flat, glabrous. Frontoclypeal suture almost straight. Eyes very slightly protruding. Antennae genus-typical, rarely exposed, usually semi-circularly folded around anterior eye margin.

*Pronotum* (Fig. 7A) 0.49–0.55 mm long (PL), 0.56–0.64 mm wide (PW), wider than long (PL/MW), widest posterior 0.25, distinctly narrower than elytra, anteriorly moderately attenuate; anterior margin distinctly convex; median carina absent, sublateral carinae present, but indistinct at posterior 0–0.2; oblique impression shallow, extending from mid lateral rim to posterior 0.2; laterobasal impression shallow; pronotal disc distinctly vaulted, very densely punctate; punctures small and shallowly impressed; setae moderately long, often broken off; anterior and anteriolateral portions sparsely punctate; interstices glabrous and flat; impressions and lateral margins rugose. Hypomeron rugose.

*Prosternum* (Fig. 7B) short; lateral portions with very dense, fine pubescence (plastron); median portion including process medially broadly impressed and glabrous; margins finely striate; prosternal process sub-pentagonal, much wider than long.

*Scutellum* (Fig. 7A) sub-cordiform, flat and glabrous.

*Elytra* (Figs 3, 7A) roundly elongate, strongly convex dorsally, 1.08–1.29 mm long (EL), ca. 1.4 times as long as wide (EL/EW), sub-parallel basal 0.1–0.5, apices separately rounded (in both sexes), with eight longitudinal, moderately to slightly impressed rows of punctures; punctures somewhat regularly arranged, much larger (1.5–2.5 times of intervals) and deeper impressed basally, very small (0.2–0.4 times of intervals) and more shallowly impressed apically; less regularly arranged in lateral rows; interstices rugose except for almost glabrous apical portion; intervals 3, 5, 7, 8 with crenulate carinae approx. from basal 0.05 to 0.75, particularly on 2^nd^ interval distinctly elevated basal 0.1–0.3; carinate intervals 5 and 7 convergent basal 0.05–0.15; all carinae with row of yellowish pubescence; lateral elytral margin serrate.

*Mesoventrite* (Fig. 7B) with deep subtrapezoidal grooves behind procoxae, medially with pair of oval impressions in males, the latter more indistinct or lacking in females.

*Metaventrite* (Fig. 7B) with irregular impressions, except for almost glabrous disc with longitudinally impression along median suture; interstices on marginal portions somewhat irregularly micro-striate to micro-reticulate.

*Abdominal ventrites* (Fig. 7C). Ventrite 1 with pair of longitudinal carinae bordering glabrous disc; ventrites 1–2 glabrous medially; lateral portions of ventrites 1–2 and almost entire ventrites 4–5 densely covered with plastron and scattered moderately long setae (Fig. 7C); apex and lateral margins of ventrite 5 more densely pubescent.

*Legs* (Fig. 3) slightly shorter than body; hind leg longest; tibia longer than tarsus and femur in all legs; coxae, femora, and tibiae moderately densely covered with short adpressed setae; inner (ventral) edge of distal tibia and tarsomeres 1–4 with fringe of long trichoid setae and short spine-like setae; outer (dorsal) edge of all femora and tibiae with longitudinal row of spine-like setae (in both sexes); apex of tibiae with pair of apical spines (most conspicuous at hind tibia). Legs not conspicuously varying between male and female.

**Figure 8.**
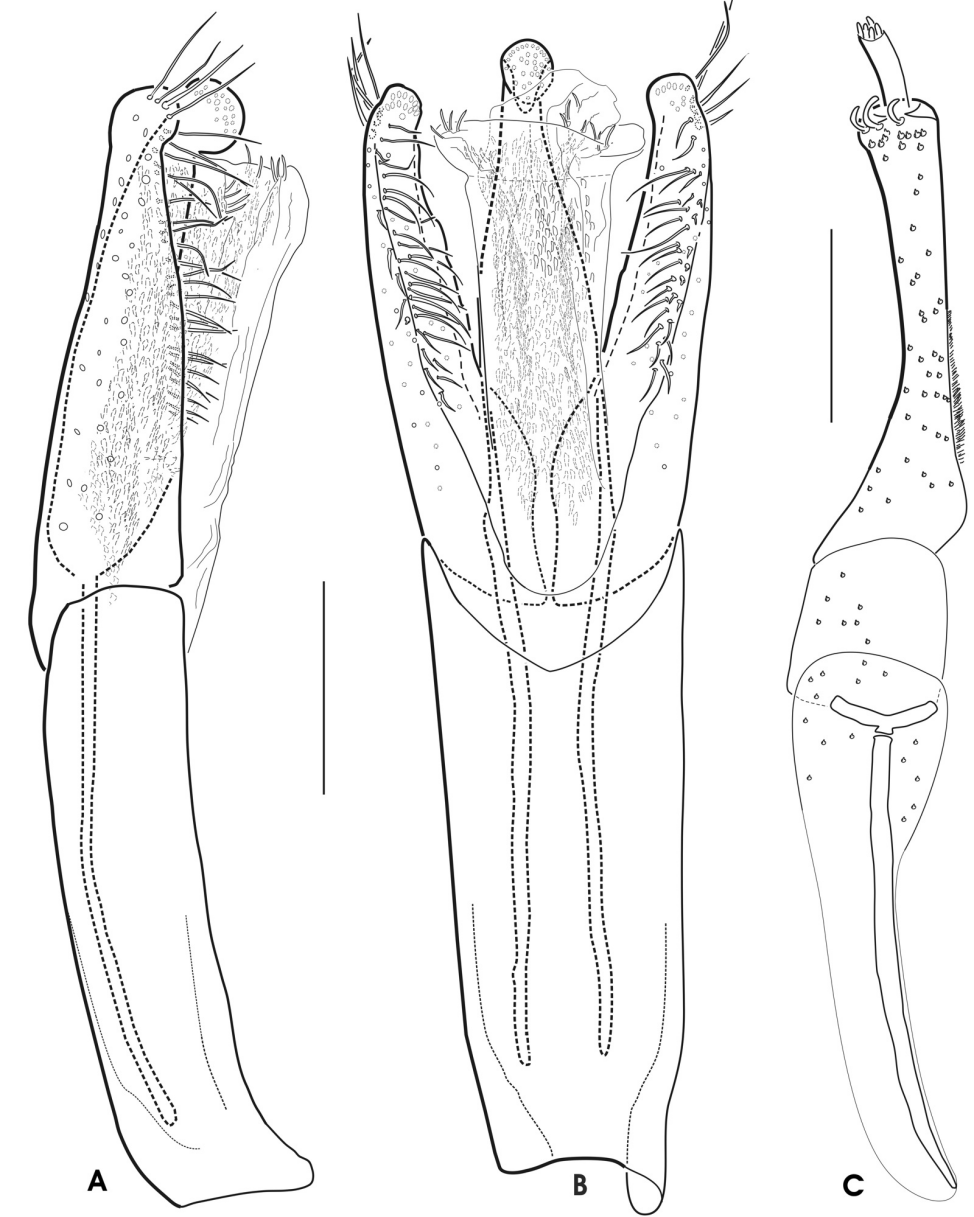
*Grouvellinus quest* sp. n. (paratype male & female from ‘NepC3g’): **A** aedeagus in lateral view **B** aedeagus in ventral view **C** ovipositor in ventral view (right half). Scale bars: 0.1 mm.

*Aedeagus* (Fig. 8A, B) 550–560 μm long, 130–150 μm wide. Phallobase slightly asymmetrical basally, reaching ca. basal 0.6 of total aedeagus length. Median lobe moderately slender, gradually tapered towards a sub-globularly expanded apex; sub-globular apex bent ventrad (lateral view). Ventral sac apically inflated internally densely stippled and with dense fringe of moderately short, thin and comparably delicate spines; few of those overreaching apical fringe. Parameres slightly shorter than median lobe, apically moderately truncate and slightly dilated ventrally (lateral view), moderately broad and evenly conical from insertion to apical 0.15 in lateral view, usually with more than 30 trichoid setae in apical half, most of them at inner face; most apical setae longest and inserted at outer surface.

*Male sternite IX* with median strut moderately long and almost rectangularly bent sub-distally; posterior portion entirely fringed with a broad, distinctly sclerotized margin; posterior margin rounded; paraprocts sub-equal in length, not reaching apical margin.

*Ovipositor* (Fig. 8C). Total length ca. 620 μm. Stylus ca. 45 μm long, slightly conical basad, very slightly bent outwards, with ca. six short sensilla. Coxite approx. half as long as entire ovipositor (ca. 300 μm), with scattered extremely short, acute setae (most densely near apex) and few apical, hook-like sensilla; distal portion medially very slender, 2.4 times as long as proximal portion, inner margin pubescent (rather inconspicuous). Valvifer almost as long as coxite (ca. 280 μm), caudal portion slightly sclerotized and with scattered, extremely short, acute setae; fibula almost straight.

##### Variability.

The paratypes from Brunei are among the smallest specimens within the given measurement ranges and have a somewhat smoother elytral surface with less impressed punctures and less elevated carinae; the aedeagus agrees well in the distinctive features (shape, size and setation of parameres; shape and size of median lobe), but the phallobase is slightly longer (0.67 of total aedeagus length), slightly slenderer and more ventrally bent basally. In specimens from either locality, elytra and pronotum are commonly incrusted with deposits making proper examination of the surface structure hardly possible.

##### Larva.

Unknown.

##### Differential diagnosis.

*Grouvellinus quest* sp. n. superficially resembles the Indonesian species *G. aeneus* (Grouvelle, 1896), but it is slightly larger (CL: 1.5–1.8 mm vs. total length 1.5–1.7 mm), black (vs. brown), and the pronotal disc is flat between punctures (vs. shagreened). Based on the only available undamaged male material of *G. aeneus* (see Jäch 1984; NMW: “Bali Baturiti D. Limnol. Exp.”) that was, however, not determined with absolute certainty, there are distinct differences in their aedeagi: larger (550–560 μm long), with longer phallobase (> half total length) and with sub-globular, ventrally bent tip of the median lobe in *G. quest* sp. n. (vs. smaller (400 μm long), with shorter phallobase (< half total length) and regularly rounded tip of median lobe in *G. aeneus*). *G. quest* sp. n. and all other new species treated in here can easily be distinguished from the only know Malaysian species *G. bishopi* by the absence of a median pronotal carina.

##### Distribution.

This species is known only from Borneo Island, namely the upper Maliau Basin in Sabah and two sites in Brunei (Fig. 11).

#### Grouvellinus andrekuipersi sp. n.

Taxon classificationAnimaliaColeopteraElmidae

http://www.zoobank.org/1AF51771-C764-489E-9122-301C688D0EFA

Figures 4 , 9 , 10

##### Type locality.

Malaysia, Sabah (on Borneo Island), Maliau Basin, upstream Giluk Falls, ca. 4°44'49"N, 116°52'38"E, ca. 950 m a.s.l. (Fig. 12A).

##### Type material.

**Holotype** ♂ (BOR/COL): “MALAYSIA: Sabah: Maliau Basin: \ upstr. Giluk Falls; bottom rock, run; \ ca. 4°44'49"N, 116°52'38"E, ca. 950m a.s.l. \ 01.X.2017, leg. I. Njunjić, CV. Pangantihon, P. Serail (GilF3g)”, terminal parts of abdomen incl. aedeagus glued separately. **Paratypes**: 2♂, 2♀ (BOR/COL) same data as holotype; 5♂, 3♀ (BOR/COL) “MALAYSIA: Sabah: Maliau Basin: \ Creek E Nepenthes Camp; bottom rock, \ run; ca. 4°43'57"N, 116°52'45"E, ca. 1000m a.s.l. \ 01.X.2017, leg. I. Njunjić, P. Serail, C. de Groot (NepC3g)”; 1♂, 1♀ (BOR/COL) “MALAYSIA: Sabah: Maliau Basin: Creek W Nepenthes Camp; bottom rock, run; ca. 4°44'04"N, 116°52'41"E, 1000 m a.s.l.; 01.X.2017, leg. I. Njunjić & H. Freitag (NepC4g)”; 2 ♂, 2 ♀ (BOR/COL) “MALAYSIA: Sabah: Maliau Basin: \ Giluk River; Cryptochorinae water plants, \ run; ca. 4°44'36"N, 116°52'21"E, ca. 980m a.s. \ l.01.X.2017, leg. I. Njunjić, H. Freitag, L. Seip, P. Piccoli (GilR2r)”; 7♂, 7♀, 7exs. (NMW) “Malaysia, Sabah, Crocker \ Range, Rafflesia Centre, \ around km 61 of road Kota \ Kinabalu, Tambunan, \ 13-14.VI. 1996, 6 a”; 1♂ (NMW) “Malaysia, Sabah, ca. 7 km S \ Sapulut, Saupi riv. in primary \ forest, ca. 500m a.s.l.,J.F. Kočiam lgt.”; 1♂, 4♀, (NMW) “Malaysia, Sabah, Crocker, \ Range, Sunsuron, 10.-11.VI. \ 1996, 8a, Sunsuron riv. flowing \ through deforested area”; 1♂, 1 ex. (NMW) “MAL., Sarawak 1993 \ Kelabit HL, Umg. Bario \ 26.2., ca 1000 m \ leg. M. Jäch (14)”; 1♂, 1 ♀, 1 ex. (NMW) “MAL., Sarawak 1993 \ Kelabit HL, 6km E Bario \ Pa Ukat, 27.2., ca 1000 m \ leg. M. Jäch (16)”; 1♂, 2exs. (SP) “MALAYSIA: Sabah: Tawau, Lucia River, 750 m a.s.l.”.

##### Etymology.

The new species is named after the Dutch astronaut André Kuipers in recognition of his engagement against the loss of the planet’s natural resources and his ambassadorship for various entomological organizations. The name was elected in an online public contest organized by the science program De Kennis van Nu of the Dutch public broadcaster NTR.

##### Description.

Body elongate obovate, 1.7–1.8 mm long (CL), 0.86–0.91 mm wide (EW), 2.0 times as long as wide (CL/EW).

**Figure 9.**
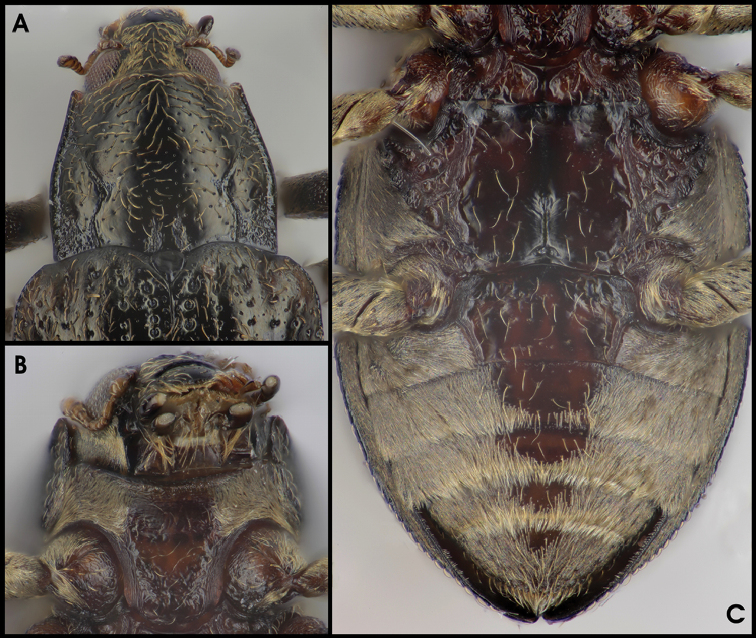
*Grouvellinus andrekuipersi* sp. n. (paratype males from ‘NepC3g’): **A** anterio-dorsal aspect with pronotum **B** anterio-ventral aspect with prosternum, meso- and metaventrite **C** posterio-ventral aspect with ventrites 1–5.

*Dorsal colouration* (Fig. 4) predominantly dark brown; pronotum black; elytra darkest at disc; basal area between shoulder and sutural interval usually with a pair of more or less distinct and extended yellowish brown spot; commonly also sub-apical elytral areas with indistinct faint paler spots; legs dark brown increasingly paler distad; tarsi and antennae golden brown; maxillary and labial palps brown; pubescence shiny yellowish. Ventral side (Fig. 9B, C) reddish dark brown.

*Head* 0.35–0.39 mm wide (HW); ID 0.16–0.17 mm; partly retractable; frons, clypeus, and anterior and lateral areas of labrum moderately pubescent; punctures small; intervals medially flat and glabrous, laterally rugulose. Frontoclypeal suture straight, indistinct. Eyes slightly protruding. Antennae genus-typical, usually semi-circularly folded around anterio-lateral eye margin.

*Pronotum* (Fig. 9A) 0.52–0.55 mm long (PL), 0.62–0.64 mm wide (PW), wider than long (PL/MW), widest posterior 0–0.3, distinctly narrower than elytra, anteriorly attenuate; anterior margin slightly convex; median carina absent, but with a pair of short posterior-median rugose patches (anterior of the scutellum); sublateral carinae very indistinct and short; oblique impression moderately deep, narrow, extending approx. anterior 0.3–0.75; laterobasal impression shallow and indistinct; pronotal disc slightly vaulted; entire pronotum moderately sparsely punctate; punctures very small and shallowly impressed; setae moderately long; interstices glabrous and flat; laterobasal impression and lateral margins rugulose. Hypomeron rugose, moderately densely pubescent.

*Prosternum* (Fig. 9B) short; lateral portions with very dense, fine pubescence (plastron); median portion including process medially broadly impressed, rugulose except for glabrous median portion; prosternal process sub-quadrate, approx. as wide as long; lateral margins finely striate.

*Scutellum* sub-triangular, medially slightly impressed, glabrous.

*Elytra* (Fig. 4) roundly elongate, moderately convex dorsally, 1.29–1.33 mm long (EL), ca. 1.5 times as long as wide (EL/EW), widest at the middle, slightly tapered anteriad; apices separately rounded, with eight longitudinal, slightly impressed rows of punctures; punctures much larger (in row 1: approx. as wide as intervals) and deeper impressed basally, increasingly smaller (in rows 1–4: 0.2 times of intervals) and more shallowly impressed apically, regularly arranged in median rows only, less regularly arranged in lateral rows; interstices glabrous; only interval 8 with (genus-typical) serrate carina; all carinae with row of yellowish pubescence; lateral elytral margin serrate.

*Mesoventrite* (Fig. 9C) with two pairs of deep sub-trapezoidal grooves, one behind procoxae, another medially, the latter more distinct in males.

*Metaventrite* (Fig. 9C) with large glabrous disc; longitudinal impression along median suture limited to posterior half; lateral portions with irregular impressions and irregularly micro-striate interstices; posterior-lateral portions with very dense, fine pubescence (plastron).

*Abdominal ventrites* (Fig. 9C). Ventrite 1 with pair of longitudinal carinae between glabrous disc and densely finely pubescent lateral portions (plastron); broad lateral portions of ventrites 1–2 and almost entire ventrites 4–5 (Fig. 9B) densely covered with plastron and scattered, moderately long setae; the latter more dense at apex and lateral margins of ventrite 5.

*Legs* (Fig. 4) approx. as long as body; hind leg longest; tibia longer than tarsus and femur in all legs; outer (dorsal) edge of all femora and tibiae with longitudinal row of robust trichoid setae (in both sexes); coxae and inner (ventral) faces of femora and tibiae densely covered with plastron-like short adpressed setae; inner (ventral) edge of distal tibia and tarsomeres 1–4 with fringe of long trichoid setae and short spine-like setae; apex of tibiae with pair of apical spines and a cluster of long spine-like setae (most conspicuous at hind tibia). Legs not conspicuously varying between sexes.

**Figure 10.**
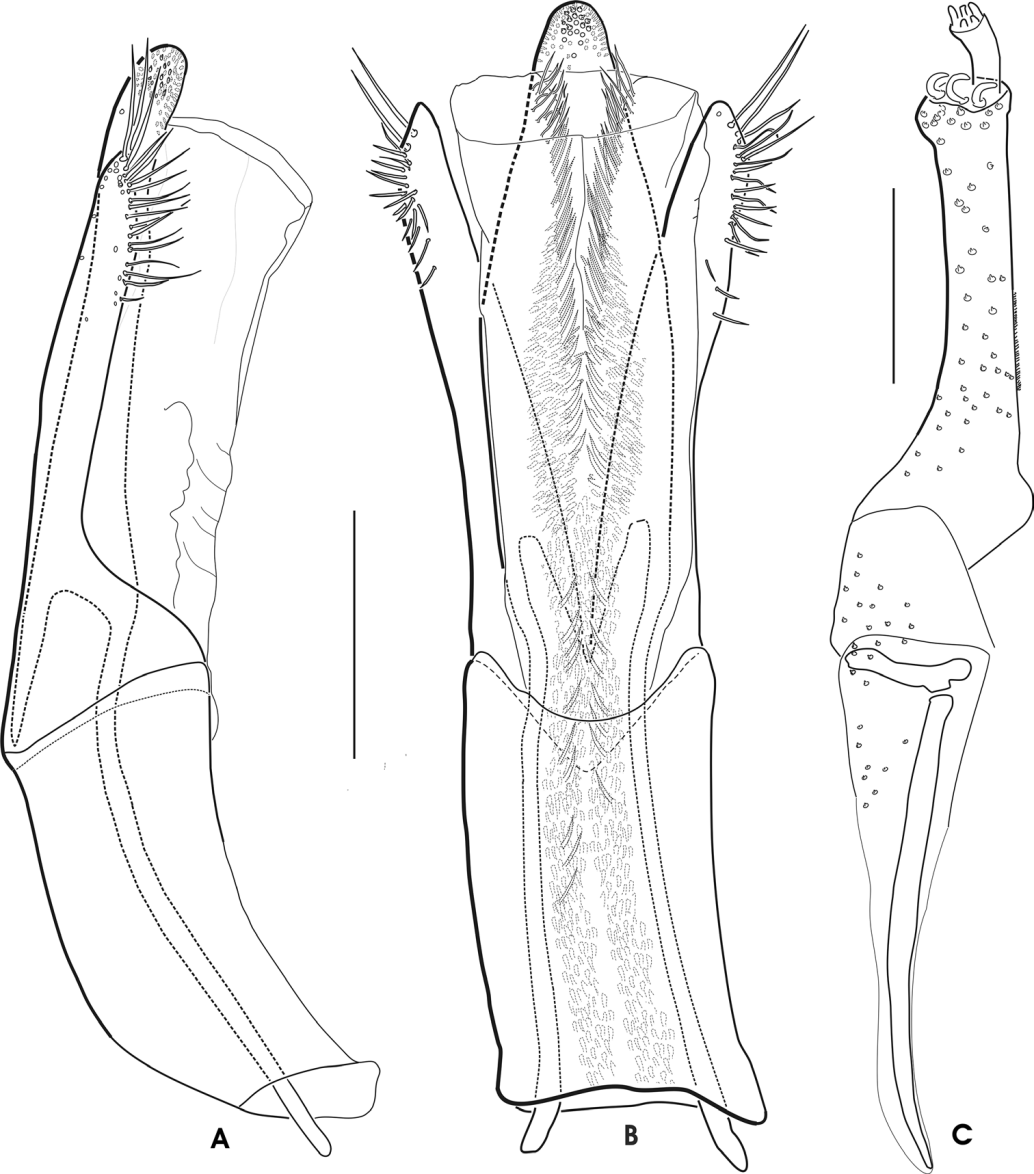
*Grouvellinus andrekuipersi* sp. n. (paratype male & female from ‘NepC3g’): **A** aedeagus in lateral view **B** aedeagus in ventral view **C** ovipositor in ventral view (right half). Scale bars: 0.1 mm.

*Aedeagus* (Fig. 10A, B) ca. 440 μm long, ca. 100 μm wide. Base reaching basal 0.46 of total aedeagus length. Median lobe ca. four times as long as wide, distinctly overreaching parameres, slightly conical towards round apex in apical third. Ventral sac apically inflated, internally densely stippled and with dense sub-median fringes of moderately long, thin spines. Parameres apically conical (in both, ventral and lateral view), very slender in apical 2/3 in lateral view, usually with more than 20 trichoid setae in apical third, most of them at outer ventral face; most apical 2–4 setae longest and inserted at dorsal face.

*Male sternite IX* as in previous new species.

*Ovipositor* (Fig. 10C) similar to that of *Grouvellinus quest* sp. n., but overall slightly shorter (total length ca. 580 μm); stylus slightly shorter (30 μm long), and more bent outwards; coxite relatively longer (260 μm long), apically more broadened, ca. 2.8 times long as proximal portion; valvifer 330 μm long.

##### Larva.

Unknown.

##### Differential diagnosis.

*Grouvellinus andrekuipersi* sp. n. is similar in size, pronotal and elytral surface structure to *G. thienemanni* Jäch, 1984 and *G. sumatrensis* Jäch, 1984, but displays a slenderer pronotum in relation to the elytra and slightly convex lateral elytral margins (vs. slightly concave or straight in basal half in *G. thienemanni* and *G. sumatrensis*). The yellowish elytral patterns commonly seen in *G. andrekuipersi* sp. n. were not observed in any examined specimen (n = 20) of the two congeners. Their entire elytra and pronotum appear overall slightly paler (brown). Additionally, the pronotal basis is entirely rugulose (“shagreened”) in *G. thienemanni* and *G. sumatrensis* (vs. glabrous with a pair of median rugose patches in *G. andrekuipersi* sp. n.). In *G. thienemanni*, the pronotal disc is additionally more densely punctate. The aedeagus of the new species is also similar in size and proportions to that of *G. thienemanni*, but in *G. andrekuipersi* sp. n., the paramere tips are distinctly conical (vs. evenly rounded in *G. thienemanni*) and the median lobe is wider and conically tapering towards apex (vs. evenly slender in apical 1/5 in *G. thienemanni*). From the previous new species (*G. quest* sp. n.), *G. andrekuipersi* sp. n. can easily be distinguished by 1) the pale elytral patches; 2) the smoother elytral surface due to the lack of any other elytral carinae than at interval 8; 3) the relatively broader and laterally convex elytra; 4) the sparse punctures of the pronotum; and 5) the smaller aedeagus with distinctly varying base, median lobe, and parameres.

##### Distribution.

**Figure 11.**
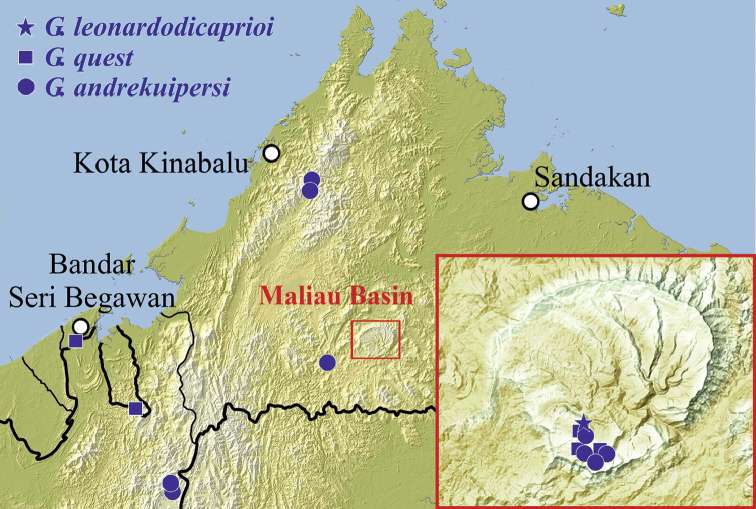
Map of the eastern tip of Borneo with large parts of Sabah, Malaysia and Negara Brunei Darussalam and the collection sites of the new *Grouvellinus* species (additional paratypes from NMW) and enlarged area of the Maliau Basin with collection sites of the first taxon expedition.

This species is known only from Borneo Island, namely the upper Maliau Basin, Tawau Hills Park, and Crocker Range in Sabah and two sites in eastern Sarawak (Fig. 11).

**Figure 12.**
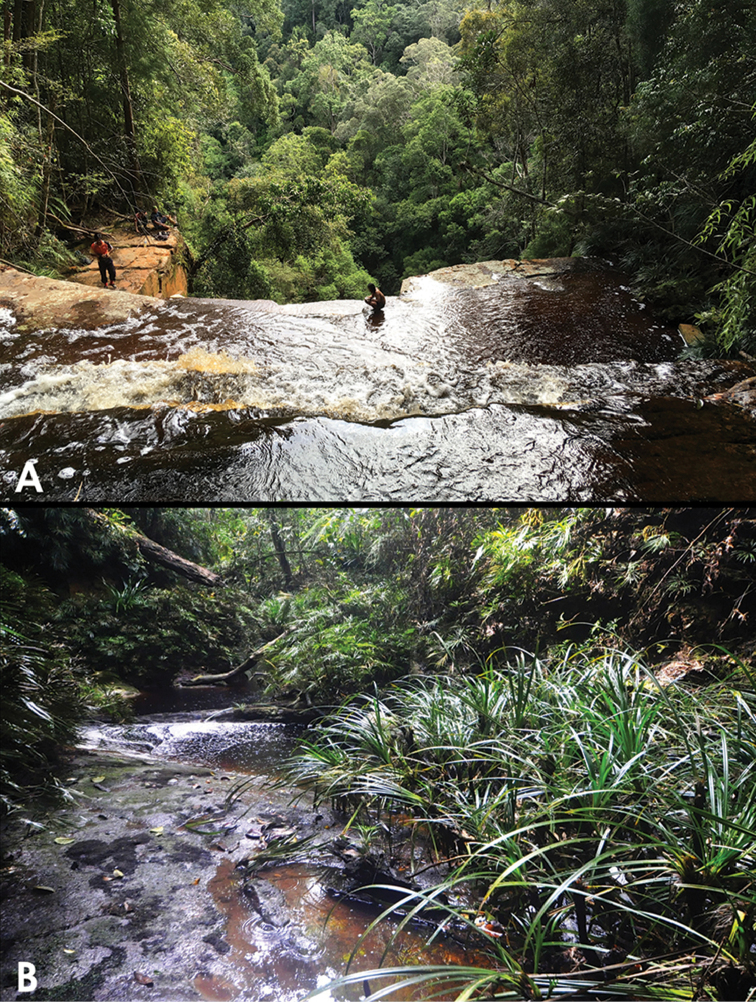
Type localities of the new species: **A** Giluk Falls (for *Grouvellinus leonardodicaprioi* sp. n. and *G. andrekuipersi* sp. n. **B** Creek east of ‘Nepenthes Camp’ (for *G. quest* sp. n.).

## Supplementary Material

XML Treatment for Grouvellinus leonardodicaprioi

XML Treatment for Grouvellinus quest

XML Treatment for Grouvellinus andrekuipersi
